# Subclinical Atherosclerosis, Hyperlipidemia and New-Onset Diabetes Should Not Be Ignored Despite Initial Angiographic Exclusion of Significant Atherosclerotic Occlusive Arterial Disease

**DOI:** 10.3390/diagnostics16152338

**Published:** 2026-07-26

**Authors:** Magdalena Wawak, Łukasz Tekieli, Marcin Piechocki, Piotr Odrowąż-Pieniążek, Magdalena Kaźnica-Wiatr, Małgorzata Mazur, Anna Maciak-Pawłucka, Monika Komar, Tadeusz Przewłocki, Anna Kabłak-Ziembicka

**Affiliations:** 1 Doctoral School of Medical and Health Sciences, Jagiellonian University Medical College, ul. Św. Łazarza, 16, 31-530 Krakow, Poland; 2 Department of Cardiac and Vascular Diseases, Institute of Cardiology, Jagiellonian University Medical College, św. Anny 12, 31-007 Krakow, Poland; 3 Department of Interventional Cardiology, The St. John Paul II Hospital, Prądnicka 80, 31-202 Krakow, Poland; 4 Department of Cardiac and Vascular Diseases, The St. John Paul II Hospital, Prądnicka 80, 31-202 Krakow, Poland; 5 Center for Innovative Medical Education, Department of Medical Education, Faculty of Medicine, Jagiellonian University Medical College, 31-008 Krakow, Poland

**Keywords:** acute coronary syndrome, atherosclerosis, cardiovascular death, cardiovascular disease, carotid intima–media thickness, carotid plaques, diabetes, major cardiac and cerebral events

## Abstract

**Background and Objectives**: Despite the presence of atherosclerosis risk factors and symptoms associated with occlusive atherosclerotic cardiovascular disease (ASCVD), up to 30% of individuals do not present significant atherosclerotic lesions at the point of diagnostic evaluation. Those in whom ASCVD was ruled out once might be believed to be at low risk of future major cerebral and cardiac events (MACCEs). Despite this, they might still have some residual risk for MACCEs. Therefore, in the present study we aimed to evaluate risk factors for atherosclerosis progression to the stage of occlusive arterial disease in patients with cardiovascular risk factors but initially free from significant ASCVD. **Materials and Methods**: Between March 2012 and February 2014, a total of 686 consecutive patients presenting with clinical suspicion of significant coronary artery disease were evaluated. The actual status of atherosclerosis burden was verified in four major arterial territories (coronary, carotid, renal, lower limb arteries) with the use of angiography, computed tomography and ultrasonography. For the present study purpose, 188 (27.4%) patients were enrolled, in whom significant ASCVD (in any of the examined territories) was ruled out. In all patients, the incidence and severity of atherosclerotic risk factors, carotid intima–media complex thickness (CIMT) and carotid plaque presence were evaluated during first hospital admission. A ten-year follow-up period for a composite endpoint of MACCEs was included. Univariate and multivariate Cox proportional hazard analyses with the Simon–Makuch method for new-onset type 2 diabetes mellitus (T2DM) were performed for MACCEs. **Results**: MACCEs occurred in 30 (16%) of 188 patients, including 5 cardiovascular deaths, 19 non-fatal myocardial infarctions, and 6 non-fatal ischemic strokes, in a median of 48 (Q1; Q3: 25; 86) months. Univariate Cox proportional hazard analysis revealed that individuals who presented with baseline LDL cholesterol > 3.2 mmol/L, CIMT > 1 mm, carotid plaque, or experienced new-onset T2DM were predisposed to MACCE with hazard ratios (HRs) of 3.21 (*p* = 0.003), 3.82 (*p* = 0.001), 5.41 (*p* < 0.001), and 36.89 (*p* < 0.001), respectively. In multivariate Cox analysis, its associations with MACCEs were retained (HRs between 2.19 and 21.47), respectively. **Conclusions**: Our findings support the need for continued risk factor optimization despite the initial exclusion of obstructive ASCVD. In this study, we identified new-onset T2DM, initial LDL cholesterol, and the presence of subclinical atherosclerosis as risk factors for MACCEs.

## 1. Introduction

Cardiovascular disease remains a leading and common cause of death in European Society of Cardiology (ESC) member countries, with over 3 million deaths per year worldwide [1]. The pooled age-standardized premature mortality rate by specific cardiovascular disease types revealed an odds ratio (OR) for coronary artery disease (CAD) of 15.57 (95% CI: 11.27, 21.5), and for stroke of 12.36 (95% CI: 8.09, 18.91) [2].

A major cause of both CAD and stroke is atherosclerosis, which is a chronic, progressive inflammatory disease of the arterial wall [3,4,5,6,7].

Despite the common belief that the presence of atherosclerosis risk factors leads inevitably to the development of occlusive atherosclerotic cardiovascular disease (ASCVD) in coronary and non-coronary arterial territories, some individuals do not present significant atherosclerotic lesions at the point of diagnostic evaluation [8,9,10]. Interestingly, those in whom ASCVD was once ruled out might be believed to represent a low-risk group for future major cerebral and cardiac events (MACCEs) [11,12]. Despite this, they might still have some residual risk for MACCEs [13,14,15,16].

The genetic and molecular mechanisms underlying atherosclerosis initiation and progression are multifactorial, including genetic, epidemiologic, and socioeconomic factors [17,18,19]. The link between atherosclerosis and its risk factors like high lipid levels, diabetes, high blood pressure values, overweight, sedentary lifestyle and exposure to cigarette smoking, although widely postulated, have diverse impacts on atherosclerosis, depending on the biomolecular mechanism activity and dynamics in an individual patient [20,21,22]. The major contributor to atherosclerosis growth, an atherothrombotic profile of lipid fractions, despite elevated levels of LDL cholesterol and/or lipoprotein, might lead to atherosclerosis at a variable progression rate [14,23,24]. In fact, there are ongoing randomized controlled studies seeking to show whether lipid-lowering therapy with statins in elderly patients with hyperlipidemia would be beneficial in primary MACCE prevention in this group of individuals [25,26]. Furthermore, elevated levels of atherogenic lipoproteins might occur early, whereas the onset of some other cardiovascular risk factors might occur at a later stage of life [27,28,29,30].

Therefore, in the present study, we aimed to evaluate risk factors for atherosclerosis progression to the stage of occlusive arterial disease resulting in MACCEs in patients with cardiovascular risk factors but initially free from significant ASCVD. We have searched for biochemical and clinical features that could be associated with the increased risk, and make them a potential diagnostic, prognostic and therapeutic target.

## 2. Materials and Methods

### 2.1. Study Population

In this single-center retrospective study, a total of 686 consecutive patients with a mean age (Standard Deviation, SD) of 62.7 (8.64) years old presenting with clinical suspicion of significant CAD were evaluated between March 2012 and February 2014. The specific inclusion criteria were age over 18 years old, presence of cardiovascular risk factors and clinical symptoms, or results of non-invasive tests suggesting ischemia in coronary artery territory. The actual status of atherosclerotic burden was verified in four major arterial territories, coronary, carotid, renal and lower limb arteries, using diagnostic tools including angiography, computed tomography, and ultrasonography.

Patients were eligible for this study if no significant atherosclerotic lesions (less than 50% lumen reduction) were observed on any of the imaging tools during the initial assessment. The other inclusion criteria were signed informed consent to participate in the study, a negative history of myocardial infarction, stroke or transient ischemic attack (no neurological symptoms present or past, and/or ischemic lesions on brain CT scans confirming the cerebral ischemia as ensured by the consulting neurologist), and no other cardiovascular ischemic events.

The exclusion criteria included significant lumen reduction of 50% or higher within at least one major arterial territory among coronary, carotid (CAS), renal (RAS) or lower extremity arterial disease (LEAD). Other exclusion criteria included other reasons for hospitalization than suspicion of significant ASCVD, hemodynamic instability, active cancer or history of valve/cardiac surgery, autoimmune disorders or chronic inflammatory disease, and lack of informed consent.

In all patients, the incidence and severity of atherosclerotic risk factors, carotid intima–media complex thickness (CIMT) and carotid plaque presence were evaluated during first hospital admission. A ten-year follow-up for a composite endpoint of fatal and non-fatal MACCE and hospitalization for symptomatic occlusive ASCVD was performed.

The study protocol was consistent with the requirements of the Helsinki Declaration and approved by the local Institutional Ethics Committee (KBET/118/B/2014 and KBET/1072.6120.47.2024). All patients signed informed consent to participate in the study.

### 2.2. Risk Factors Assessment

On admission, all patients underwent evaluation for cardiovascular risk factors. Five classic cardiovascular risk factors were assessed for presence, including arterial hypertension, hyperlipidemia, underweight and overweight or obesity, type 2 diabetes mellitus (T2DM), and smoking at study entry. Body mass index (BMI; the weight in kilograms divided by the square of the height in meters) was calculated for all study participants. Underweight was defined as a BMI of less than 20, and overweight or obesity was defined as a BMI of 25 or more. Arterial hypertension, T2DM, and hyperlipidemia were determined on the basis of medical history, participant report, or new diagnosis at baseline examination using measures dependent on the standard operating procedures. The definitions of cardiovascular risk factors were adopted from the respective scientific European Society of Cardiology statements [31,32,33,34]. The prevalence of conventional risk factors (T2DM, hyperlipidemia, arterial hypertension, and smoking current or previous) was recorded.

In all patients, baseline total cholesterol, low-density lipoprotein cholesterol (LDL-cholesterol),high-density lipoprotein cholesterol (HDL-cholesterol), high-sensitivity C-reactive protein (hs-CRP), renal function, glycated hemoglobin (HbA1c) and glucose levels were measured from fasting blood samples.

### 2.3. CIMT and Carotid Plaque Assessment

High-resolution B mode, color Doppler, and pulse Doppler ultrasonography of both carotid arteries was performed with an Toshiba Aplio PowerVision ultrasound machine (Toshiba Medical Systems Co., Ltd., Tokyo, Japan) equipped with 4–11 MHz linear array transducer. Patients were examined in the supine position with the head tilted backward. After the carotid arteries were located by transverse scans, the probe was rotated 90° to obtain and record a longitudinal image of the anterior and posterior walls. The maximum CIMT or plaque diameter (whichever applicable) was measured at the near and far walls of the common carotid artery, the bifurcation, and the internal carotid arteries, and was expressed as a mean aggregate value. Carotid atherosclerotic plaques were defined according to the Mannheim consensus criteria: (1) a focal structure encroaching into the arterial lumen by at least 0.5 mm, (2) a lesion protruding ≥ 50% beyond the surrounding intima–media thickness, or (3) a focal thickening > 1.5 mm measured from the media–adventitia interface to the intima–lumen surface [14,35].

The mean of maximum plaque diameter or CIMT thickness was assessed and included in further analysis. Results were presented as either mean CIMT value or plaque presence.

### 2.4. Follow-Up—Cardiovascular Events

The mean follow-up period was 113 ± 33 months (range 9–120 months). No patient was lost to follow-up. Incident MACCE was defined as a first fatal or nonfatal myocardial infarction (MI), ischemic stroke (IS) associated with carotid artery disease, or death from a cardiovascular cause.

Cardiovascular death was defined as fatal IS, fatal MI, or other cardiovascular death (i.e., any sudden or unexpected death [while sleeping] unless proven as non-cardiovascular on autopsy). Ischemic stroke had to be sourced from a neurologist or hospital files to ensure a reliable diagnosis. Lesion progression was defined as an increase in lesion stenosis to ≥50% requiring revascularization, in a location with no prior stenosis (<50%).

### 2.5. Statistical Analysis

Qualitative data were presented as numbers with percentages [*n* (%)], and groups were compared using the Pearson’s chi-square test or Fisher’s exact test. The normality of quantitative data distribution was investigated using the Shapiro–Wilk test, and the homogeneity of variance was assessed using the Levene test. Due to their normal distribution, all quantitative data were shown as the mean with standard deviation [mean (SD)] and compared using the Student’s *t*-test.

The optimal LDL-cholesterol cutoff value for predicting the composite endpoint was determined using ROC curve analysis and Youden’s J statistic. Cumulative incidence curves for the composite endpoint were constructed using the Kaplan–Meier estimator, and group comparisons were made using the log-rank test. For the time-dependent covariate (incident diabetes mellitus type 2), cumulative incidence curves were constructed using the Simon–Makuch method, and differences between groups were evaluated using the Mantel–Byar test.

Univariate Cox proportional hazards analysis was performed to determine the risk factors for the composite event. Incident diabetes was incorporated into the Cox models as a time-dependent covariate. The hazard ratio (HR) and 95% Confidence Intervals (95% CI) were calculated for these factors. The independent MACCE predictors, variables demonstrating statistical significance in the univariate analysis (*p*-value < 0.1), were subsequently included in the multivariate Cox proportional hazards models using the enter method, with a maximum of three variables entered simultaneously. Closely related variables were not included simultaneously in the multivariate models, resulting in models including CIMT or carotid plaque separately.

The significance level α was set at 0.05. Survival analyses were carried out using PS IMAGO PRO 10.0 and R software (version 4.6.1) within the RStudio environment (version 2026.06.0).

## 3. Results

### 3.1. Baseline Study Group Characteristics

**Figure 1:**
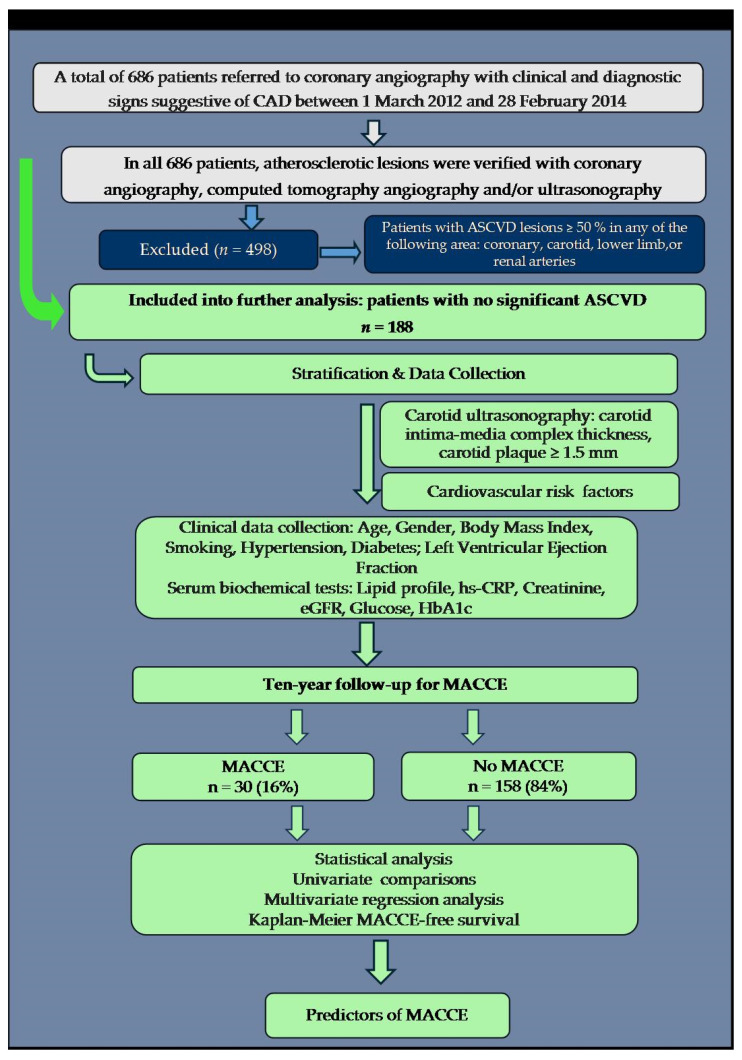
Study flow chart depicting initial study cohort, enrollment and exclusion criteria, key variables measured and cardiovascular outcomes.

As a result of diagnostic workups, a total of 498 patients were diagnosed with significant lumen reductions of 50% or higher within at least one major arterial territory among coronary, carotid, renal and/or lower extremity arterial beds. These patients were excluded from this study. The study flowchart is presented in Figure 1.

A total of 188 (27.4%) patients were enrolled in the present study, in whom significant ASCVD (in any of the examined territories) was ruled out. Upon enrollment, the patients’ mean age (SD) was 62.7 (8.42) years old, ranging between 42 and 80 years old. Males and females accounted for approximately 65% and 35% of study participants. Arterial hypertension, T2DM, smoking, and hyperlipidemia were diagnosed in 73.4%, 20.7%, 35.6% and 76.1% of individuals, respectively. The mean (SD) number of cardiovascular risk factors was 2.77 (1.16). The baseline group characteristics are presented in Table 1.

At hospital discharge, 130 (69.1%) patients were given ACE inhibitors, 25 (13.3%) angiotensin receptor blockers (ARBs), 64 (34%) β-blockers, 146 (77.6%) calcium channel blockers, 136 (72.3%) statins, 3 (1.6%) fibrates, 72 (38%) diuretics, and 84 (44.6%) aspirin. Among patients with T2DM, insulin was given to 4 (10.3%), sulfonylurea to 21 (53.8%) and metformin to 35 (89.7%) patients.

### 3.2. Cardiovascular Outcomes and Cardiovascular Risk Factors Distribution

A total of 188 patients with no significant ASCVD were followed up for MACCE. During a ten-year follow-up period, MACCE occurred in 30 (16%) patients. There were five cardiovascular deaths, six non-fatal ISs related to carotid artery atherosclerotic occlusive disease, and nineteen non-fatal acute MI episodes managed with primary percutaneous coronary interventions. MACCE occurred in a median time of 48 months (Q1 and Q3, 25 and 86, respectively).

Individuals with MACCE, compared to non-MACCE, were slightly older at baseline, and were more likely to have more cardiovascular risk factors at entry (2.97 ± 1.22 vs. 2.73 ± 1.15); however, neither reached statistical significance (*p* = 0.7190, *p* = 0.317, respectively)—Table 1.

Hyperlipidemia was more prevalent in MACCE compared to the non-MACCE group (93.3% vs. 72.8%, *p* = 0.016). There was no statistically significant difference concerning gender distribution (*p* = 0.337) and prevalence of abnormal BMI (*p* = 0.898). The prevalence of arterial hypertension and smoking at study entry was slightly higher in the MACCE group compared to the non-MACCE group; however, these differences did not reach statistical significance (*p* = 0.659, *p* = 0.169, respectively)—Table 1. The frequency of T2DM in MACCE vs. non-MACCE individuals was similar in both groups at study entry (*p* = 0.913).

MACCE occurred more frequently in patients with higher baseline levels of LDL-cholesterol (3.47 ± 1.11 mmol/L vs. 3.08 ± 0.92 mmol/L; *p* = 0.0438) and lower eGFR (*p* = 0.037). There were no statistically significant differences regarding baseline levels of fasting glucose (*p* = 0.754), HbA1c (*p* = 0.215), creatinine (*p* = 0.089), total cholesterol (*p* = 0.2387), HDL-cholesterol (*p* = 0.8177), triglycerides (*p* = 0.8205) and hs-CRP (*p* = 0.3616), in patients with MACCE compared to the non-MACCE group. The baseline left ventricular systolic function fraction on echocardiography was also similar (*p* = 0.357) between both groups.

Statistical analysis revealed that individuals who suffered from MACCE at follow-up had higher CIMT values at baseline (1.160 ± 0.273 mm vs. 0.958 ± 0.209 mm, *p* < 0.001) and were more likely to have CIMT thickening > 1.0 mm (76.7% vs. 36.1%, *p* < 0.001) and carotid plaque, defined according to the Mannheim consensus criteria (73.3% vs. 29.7%, *p* < 0.001), compared to individuals with non-MACCE at follow-up.

During the follow-up period, a new onset T2DM was identified in 14 (7.4%) patients, including new incidences in 3 (1.9%) patients in the non-MACCE group and 11 (36.7%) patients in the MACCE group (*p* < 0.001). In all cases of MACCE but one, T2DM recognition preceded MACCE incidence. In one case, T2DM was diagnosed together with MACCE incident.

### 3.3. Risk Factors of MACCE in Univariate and Multivariate Cox Proportional Hazard Analysis

The results of the univariate and multivariate Cox proportional hazard analyses for MACCE are presented in Table 2.

Univariate Cox proportional hazard analysis (Table 2) revealed that individuals who were presented with hyperlipidemia at baseline, initial CIMT thickening above 1.0 mm, and carotid plaque at study entry were more likely to suffer from MACCE with hazard ratios (HR) of 4.89 (95% Confidence Interval, CI: 1.17–20.5; *p* = 0.03), 3.82 (95% CI: 1.70–8.58; *p* = 0.001), and 5.41 (95% CI: 2.10–10.97; *p* < 0.001), respectively. Interestingly, according to CIMT quartiles, only patients with CIMT values in the highest quartile compared to the lowest quartile showed significant increases in MACCE risk (HR—6.30, 95% CI—1.43–27.72; *p* = 0.015) (Table 2), which demonstrates non-linear association. Baseline LDL-cholesterol, as a continuous variable, was significantly associated with an increased risk of MACCE (HR—1.04, 95% CI—1.01–1.08; *p* = 0.025). A new onset T2DM was associated with a 36.89-fold increase in risk of MACCE (95% CI: 4.67–23.1; *p* < 0.001), compared to patients without diabetes (Table 2).

The ROC analysis revealed that the optimal cut-off for LDL-cholesterol level as a MACCE predictor was >3.2 mmol/L, with a sensitivity of 70%, a specificity of 61%, and an AUC of 0.64 (95% CI: 0.53–0.75).

Further statistical analysis revealed the significant collinearity of variables “plaque” and CIMT with an AUC of 0.84 (95% CI: 0.77–0.92); *p* < 0.001. Therefore, a multivariate Cox proportional hazard analysis was performed in two separate models, one with the inclusion of plaque presence (Model 1), and the other including CIMT > 1.0 mm (Model 2).

In Model 1, multivariate Cox proportional hazard analyses, including cross-sectional analyses of cardiovascular risk factors at and after study entry, showed that the risk of MACCE was higher for individuals with new onset time-adjusted T2DM (HR—18.62; 95% CI—7.97–43.5; *p* < 0.001), LDL-cholesterol levels above 3.2 mmol/L (HR—2.19; 95% CI—0.99–4.94; *p* = 0.05), and plaque presence at study entry (HR—4.02; 95% CI—1.72–9.36; *p* = 0.001).

In Model 2, a baseline CIMT above 1 mm was an independent risk factor for MACCE (HR—2.89; 95% CI—1.27–6.59; *p* = 0.012). Additionally, a new onset time-adjusted T2DM and LDL-cholesterol levels above 3.2 mmol/L retained associations with MACCE, with HRs of 21.47 (95% CI—9.33–49.40; *p* < 0.001) and 2.90 (95% CI—1.32–6.59; *p* = 0.012), respectively.

### 3.4. Kaplan–Meier Event-Free Survival Depending on Carotid Subclinical Atherosclerosis and Type 2 Diabetes Mellitus Presence

**Figure 2:**
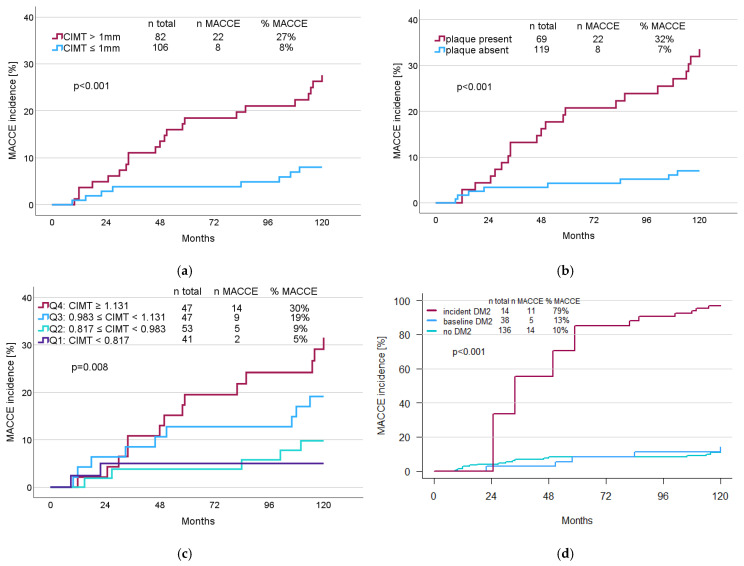
Cumulative incidence curves for MACCE using the Kaplan–Meier estimator (**a**–**c**), (**a**) via carotid intima–media complex thickening (>1 mm) at study entry; (**b**) via carotid plaque presence at study entry; (**c**) via baseline CIMT quartiles; and (**d**) the Simon–Makuch method via the time-adjusted onset T2DM status. n, number.

For baseline CIMT, higher MACCE risk was seen in categories of carotid intima–media complex thickening (CIMT value > 1 mm) vs. normal CIMT value (CIMT < 1.0 mm), with cumulative MACCE rates at 10-year follow-up of 27% and 8%, respectively (Figure 2a, upper left panel). For carotid plaque presence, cumulative MACCE incidence was 32% compared to plaque absence (7%) (Figure 2b, upper right panel).

Stepwise higher risks were seen in categories of carotid intima–media complex quartiles (Q1—CIMT value < 0.817 mm; Q2—CIMT between 0.817 and 0.982; Q3—between 0.983 and 1.330 mm; Q4—CIMT equal to or higher than 1.331), with cumulative MACCE rates of 5%, 9%, 19% and 30%, respectively (Figure 2, lower left panel).

Finally, stepwise higher risks were seen in categories of absent diabetes mellitus, T2DM observed at study entry, and a new onset T2DM during the follow-up period, with cumulative MACCE rates at 10 year follow-up of 10%, 13%, and 79%, respectively (Figure 2, lower left panel).

## 4. Discussion

### 4.1. Clinical Significance of Our Findings

The maintenance of tight cardiovascular risk factors control in patients with primarily excluded significant lesions in arterial territories remains a challenging task [36,37]. A normal angiogram may lead a patient to a false feeling of being secure from cardiovascular event risk [36]. Consequently, reduced patient’s motivation, together with lesser attention to cardiovascular risk factor control, eventually leads to atherosclerosis progression, which is a natural course of unsupervized thrombo-atherosclerotic processes [38,39,40]. In a vast number of patients, genetic, inflammatory and social predispositions come to the forefront, unless strictly managed [41,42,43]. Consequently, deprescribing key pharmacological medications, primarily including statins and blood-lowering agents in the context of normal arteries, delayed intervention in patients with hyperglycemia, and inevitably put patients at future cardiovascular risk [44,45,46].

The observational studies demonstrate that diagnostic work-up results can have clear and immediate effects on how patients’ view and emotionally respond to their symptoms [47,48]. Personal beliefs about an individual’s health condition tremendously impact medical attitudes, subjective norms and medication adherence. Compliance is better in well-motivated individuals with confirmed cardiovascular disease compared to individuals in whom arterial complications were excluded at the point of diagnostic work-ups [48]. A diagnosis of non-obstructive CAD or normal cardiac tests leads to a lower likelihood of following recommended lifestyle changes or medication regimens designed for management [9].

This phenomenon is also well-known in studies on cardiovascular risk factor management [49,50,51]. In a study by Hagger et al., attitudes (β = 0.331, *p* < 0.001), subjective norms (β = 0.121, *p* = 0.009), and beliefs about medication overuse (β = −0.160, *p* < 0.001) were significant predictors of intentions to take medication in familial hypercholesterolemia [49,50]. Similarly, in hypertensive individuals, the perception of concern about the disease has a significant impact on adherence to the treatment regimen [51].

In light of the aforementioned evidence-based data, the results of our current study are particularly important. Firstly, we have shown a high exclusion rate of occlusive ASCVD. Of a total 686 consecutive patients admitted with suspected significant ASCVD, 188 (27.4%) patients were ruled out. This is in line with findings from cardiac catheterization centers worldwide [52,53,54,55]. The averaged reported frequency of negative tests for obstructive CAD accounts for 24–41% of individuals following coronary angiography or contrast-enhanced coronary-computed tomography angiography [52,53].

Researchers’ opinions on whether repeated angiographic studies should be considered at a later period are a point of debate [52,53,54,55]. Some argue that once excluded, occlusive CAD is unlikely to progress to severe disease [54], whereas others report a high progression rate of coronary arteriosclerosis [55,56]. In a study by Rowe et al., no patient developed any significant (>50% stenosis) disease; in 11%, mild progression was observed (average 37% stenosis), while 89% continued to have normal arteries at a median 51 months [53]. Similarly, Androshchuk et al. reported that previously normal coronary angiography and minor CAD are unlikely to progress to significant disease and the incidence of MI (<0.5%) in these patients at 5 years [54]. In the aforementioned study, no change in disease progression in 79.4% of patients was noted [54]. Conversely, in the remaining 20.6% of patients, ‘normal’ coronary arteries progressed to minor CAD in the follow up studies at a median follow up of 6.8 years [54]. Therefore, the previous view was that the disease progressed very slowly throughout life.

On the contrary, in a study by Roguin et al., 3% of patients underwent percutaneous coronary intervention due to CAD progression over a median time of 4 years [55]. The PESA study enrolled 4184 asymptomatic middle-aged participants who underwent a noninvasive imaging assessment of multi-territorial subclinical atherosclerosis [56]. The PESA study demonstrated that over a short follow-up of just three years, 40% of individuals aged between 40 and 50 years showed the major progression of atherosclerosis in distinct locations, including the carotid, femoral, and coronary arteries [56]. Future data from the PESA study will show whether this progression is associated with risk of MACCE. Bertolini et al. demonstrated a MACCE incidence of nearly 50% in a total of 744 patients who initially presented with type 2 (non-coronary plaque rupture) MI over a 53-month follow-up period [57]. In this study, the authors indicated distinct differences in MACCE rates depending on MI phenotypes. Phenotype 1 patients were younger and healthier, while phenotype 2 patients were older with greater comorbidity and coronary atherosclerotic burden, resulting in MACCE rates of 23.8% and 60.8% (*p* < 0.001), respectively [57]. Thus, significantly different baseline clinical characteristics and precipitating factors determined long-term outcomes and prognostic factors, despite similar management strategies applied in both groups.

In our current study, despite primarily “normal” arteries on diagnostic work-ups, MACCE occurred in approximately one out of six (16%) patients, including progression of CAD in 65% of patients, during a ten-year follow-up period. Of note, those patients presented with a similar baseline distribution of cardiovascular risk factors and similar biochemical characteristics, including mean age and BMI, gender distribution, and the prevalence of T2DM and hypertension. At study entry, MACCE vs. non-MACCE patients differed significantly with regard to hypercholesterolemia prevalence, eGFR and LDL-cholesterol level, whereas serum creatinine, glycemia and hs-CRP levels were similar. The prevalence of hyperlipidemia and arterial hypertension was high at entry, allowing patients to be offered optimal treatment. A multivariate Cox proportional hazard analysis revealed that individuals who presented with higher baseline LDL-cholesterol (above 3.2 mmol/L), CIMT > 1 mm, and carotid plaque, or who developed new onset T2DM, were more likely to suffer from MACCE, with an approximate 2-to-21-fold risk increase.

Ultimately, in the present study, we have demonstrated the importance of surveillance in three major areas: (1) optimization of LDL-cholesterol control; (2) importance of baseline carotid alterations such as carotid intima–media complex thickening and carotid plaque presence; and (3) an additive and overwhelming risk of MACCE in patients developing T2DM.

### 4.2. Importance of Hypercholesterolemia

We have demonstrated that the initial presence of hypercholesterolemia, along with higher LDL-cholesterol levels, remains a vital and important contributor to future cardiovascular ischemic events. Traditionally, the main drivers of atherosclerosis initiation are considered to be elevated levels of LDL-cholesterol, with impaired reverse cholesterol transport to the liver due to decreased levels of HDL-cholesterol [23,58]. This process involves very complex mechanisms, including macrophage activity and microRNA expression engaged in lipid metabolism (like miR-33a/b, miR-155, miR-146), in line with the liver steatosis leading to impaired liver lipid clearance [59,60,61]. Macrophages are involved in cholesterol homeostasis and are regulated by several miRs involved in lipid metabolism [62]. In cholesterol homeostasis, miR-33a increases plasma LDL-cholesterol and promotes the progression of plaque burden, while miR-33b increases plasma HDL-cholesterol levels and promotes reverse cholesterol transport to the liver [63]. Additionally, the miR-33 family impairs insulin signaling by repressing genes (like sirtuin-1) that are important for maintaining insulin sensitivity, cellular energy balance, and the mitochondrial function, while exaggerating inflammatory pathways and increasing obesity risk in individuals on a high-fat diet [62].

Both miR-33a and miR-33b increase cholesterol accumulation in the liver, resulting in the progression of metabolic liver diseases [63]. Furthermore, the members of the miR-33 play a pivotal role in fatty acid oxidation, which is critical for the transport and breakdown of long-chain fatty acids in the mitochondria. By repressing these genes, miR-33 reduces the capacity for fatty acid oxidation, thereby promoting lipid accumulation in the liver. Conversely, miR-33 inhibition is considered to enhance fatty acid utilization and impact steatosis treatment. Notably, miR-33a knockout in hepatocytes reduces steatosis with improved insulin sensitivity and glucose homeostasis [63].

From this perspective, Metabolic Dysfunction-associated Steatotic Liver Disease (MASLD) is central contributor to atherosclerosis progression. Thus, an individuals’ ability to maintain liver function at a sufficient level might be a key factor associated with the rate of atherosclerosis progression [64]. Accordingly, effective lipoprotein-lowering treatment is pivotal in lipid metabolism regulation and insulin sensitivity.

Major clinical trials suggest that statins can offer an average 28% reduction in LDL cholesterol and a 31% reduction in relative risk, leaving a residual risk of about 69% [20].

Therefore, the residual risk remaining after statin treatment is greater than the risk that is eliminated. Of note, several polymorphisms relating to cholesterol metabolism and response to statins have been identified, limiting statins’ effectiveness [65,66]. As a result, the identification of additional risk factors and the introduction of other medications to reduce residual risk, particularly those administered together with statin drugs, have been an ongoing quest [20].

The present study model is LDL-centric, whereas the importance of other lipoproteins, such as APO B, lipoprotein (a), triglycerides, low-HDL cholesterol and mutations in genes for cholesterol receptors, should be considered as well [14]. The major evidence gathered up today concerns coronary heart disease. According to the studies, when LDL cholesterol is controlled, the odds of CAD fall by about 40% per 0.194 mmol/L (7.5 mg/dL) rise in HDL cholesterol [67]. The odds of CAD increase by approximately 20% per 0.260 mmol/L (23 mg/dL) rise in triglyceride levels. This translates to a fall in MACCE by about 1.1% for each 0.026 mmol/L (1 mg/dL) rise in HDL cholesterol when LDL cholesterol is lower than or equal to 1.8 mmol/L (70 mg/dL) [68].

The introduction of novel agents, such as PCSK9 inhibitors, evolocumab and alirocumab, lowers LDL cholesterol by 45–58% when used in combination with maximally tolerated statins [69,70]. Inclisiran lowers LDL-C on average by 48–52% [71]. The ODYSSEY Outcomes (alirocumab) and FOURIER (evolocumab) trials both showed a 15% relative risk reduction (1.5% to 1.6% absolute risk reduction) compared to placebo when added to statin therapy [72]. The additive advantage of PCSK9 inhibitors relies on the increase in LDL receptor density on the liver surface and a reduction in serum LDL cholesterol levels through two concurrent mechanisms, namely, with the use of monoclonal antibodies (PCSK9i-Ab) and with small interfering RNA (siRNA) [1,73].

In line with this, targeting miR-33 represents a promising yet intricate strategy for the treatment of cardiovascular and metabolic diseases.

Last but not least, lipoproteins may contribute to atherogenesis independently of LDL oxidation through mechanisms such as the activation of inflammasomes, similarly to viral myocarditis models and/or glycation [42,43,74]. In particular, the glycation and modification of LDL cholesterol by methylglyoxal, a reactive dicarbonyl metabolite, is highly pro-atherogenic [20]. Furthermore, atherosclerosis progression is characterized by a persistent elevation in circulating pro-inflammatory cytokines (such as IL-6, TNF-α, and CRP), the activation of innate immune pathways (e.g., the NLRP3 inflammasome), and the dysregulation of both adaptive and innate immunity [75]. The process of inflammation not only contributes to atherosclerosis itself, but also interacts with cardiovascular risk factors, amplifying their adverse cardiovascular effects [76].

### 4.3. Impact of Arterial Hypertension on Atherosclerosis Initiation and Progression

The role of the elevated arterial blood pressure values should not be neglected in the pathogenesis of atherosclerosis and its progression [77]. Early changes to the arterial wall resulting from increased blood pressure lead to arterial wall stiffening due to collagen fiber production and accelerated elastin fiber degradation [78]. This, in turn, further increases arterial blood pressure [78]. Chronic high blood pressure leads to arterial wall damage through mechanical stress, endothelial dysfunction, increased inflammation, oxidative stress, and renin–angiotensin–aldosterone system (RAAS) activation [79]. Stiffened arteries struggle with blood pressure changes, raising systolic blood pressure and pulse pressure. Consequently, increased systolic pressure further hardens arteries, creating a harmful cycle of inflammation and calcification [78,79]. Elevated blood pressure is a major risk factor for severe medical conditions, including arterial ischemic events (myocardial infarction, both hemorrhagic and ischemic stroke), hypertensive crises, atrial fibrillation, Alzheimer’s disease and vascular dementia, renal and heart failure, and many others [80]. Thus, decreasing blood pressure to the optimal values exerts a protective role in atherosclerosis, and the blood-lowering therapy is one of the cornerstones of atherosclerosis attenuation and the lowering of the risk of MACCE [80]. The first-choice blood-lowering agents include calcium channel blockers, given together with angiotensin-converting enzyme inhibitors (ACEIs) or angiotensin receptor blockers (ARBs) [81]. All of them have anti-atherosclerotic potential, and act on the blood vessels, mainly by reducing the smooth muscle tone of the vascular wall, which is particularly beneficial in the elderly [82].

However, life-long adherence to antihypertensive medication, especially in free-dose combinations, imposes a significant challenge [83,84]. In a study by Perrone et al., only about half of the patients who were prescribed the free combination of amlodipine, perindopril and atorvastatin maintained a Proportion of Days Covered of 80% or greater, and a significant portion required dosage modifications to manage their conditions effectively [84]. On the contrary, the administration of fixed-dose co-formulations such as statins and blood-lowering agents in one pill, significantly improves adherence, and results in a greater proportion of patients achieving treatment targets [85].

In conclusion, adherence to statin therapy, in combination with other pharmacological therapies for other cardiovascular risk factors like arterial hypertension, is crucial to improving prognosis and reducing the contribution to atherosclerosis initiation and progression.

### 4.4. New Onset Type 2 Diabetes Mellitus

In the present study, we have observed nearly 80% MACCE incidence in patients who developed T2DM during the observational period, compared to 10% MACCE incidence in patients free from diabetes. The connection between T2DM and risk of MACCE might not be surprising; however, the dimension of cardiovascular risk imposed by a new-onset T2DM might be challenging.

Patients with diabetes have a greater risk of MACCE and worse clinical outcomes after the event than their counterparts without the disease [20]. About 30% of patients with diabetes experience rapid CAD progression, which is even faster in those who already have subclinical atherosclerosis and/or coronary heart disease. In a study by McMullan et al., among 582 patients with a normal coronary arteries angiogram, the five-year cardiovascular death rate was 7.7%, with diabetes as an independent predictor of cardiovascular events (2.98, 95% CI, 1.20–7.41) [86].

In the Multi-Ethnic Study of Atherosclerosis (MESA), the highest calcium score and atherosclerosis progression were noted in patients with T2DM and metabolic syndrome at both the 2.4- and 4.9-year follow-up coronary calcium CT scans [87]. In line with this, the calcium score progression rate was a good predictor of MACCE [87].

Thus, special attention should be paid to glycemia control, as well as to pre-diabetic patients, as hyperglycemia constitutes a concurrent metabolic pathway apart from LDL–HDL lipoproteins’ homeostasis, leading to the fast progression of atherosclerotic lesions [88]. Among many other things, oxidative stress and mitochondrial dysfunction, the excess release of free fatty acids from adipose tissue, insulin resistance, lipoprotein changes, the lower activity of lipoprotein lipase associated with insulin resistance, the hepatic over secretion of triglyceride-rich VLDL and the impaired clearance of these particles, in association with higher plasma levels of ApoC-III, are central mechanisms connecting T2DM, inflamed adipose tissue and cardiovascular risk [88].

The need for the timely identification of a new onset T2DM as a risk factor for atherosclerosis progression arises from concurrent mechanisms of atherosclerosis growth initiation and progression, such as glycation, lactylation, the NLRP3 pathway, new microRNA expression and the intensification of oxidation, which imposes an additive burden on lipid metabolism [89,90,91,92,93]. This “metabolic transition” in T2DM accelerates vascular inflammation and endothelial dysfunction, promoting the rapid progression of subclinical lesions to occlusive disease. As a result, patients with T2DM are less likely to achieve the adequate control of lipid levels with statins compared to counterparts free from T2DM [94].

Our study indicates slightly higher baseline values of serum glucose levels in patients with future MACCE. We believe that high–normal levels of serum glucose should be carefully monitored, as T2DM might be diagnosed later in these patients. New-onset T2DM should heighten physicians’ attention to possible atherosclerosis progression. This indicates the importance of the identification of high–normal fasting glucose levels and close surveillance for overt diabetes. Mader et al. observed that T2DM contributes tremendously to MACCE, irrespective of initial atherosclerosis extent (HR, 1.14; 95% CI, 0.86–1.52), accounting for 19.1% in patients without atherosclerosis at study entry, compared to 34.1% in those with minor atherosclerosis, and 58.1% in those with major atherosclerosis during a 7 year follow-up period [95]. In line with this, T2DM was identified as an independent risk factor for MACCE (OR, 1.61; 95% CI, 1.31–1.98) [95].

Despite its widespread clinical use, HbA1c is most often interpreted using fixed diagnostic thresholds, which may not fully capture the biological complexity of metabolic processes [40,96,97]. A key finding of a recent study was the identification of non-linear risk patterns linking HbA1c to complication susceptibility [96,97]. The probability of presenting at least one complication has begun to increase at HbA1c levels below the diagnostic threshold for diabetes, with a noticeable inflection within the 5.5–6.4% interval [97].

Altogether, T2DM occurs broadly at later stages of life [80]. The clinical significance of high–normal values of fasting glucose and HbA1c is high when no glucose-lowering therapy except for diet counseling is introduced. Clinical scenarios might differ nowadays, due to the large introduction of flozins and GLP-1 receptor agonists given for indications apart from glucose levels, including heart failure, nephroprotection and weight reduction in overweight and obese patients [28,98].

In particular, the cardiovascular-protective properties of GLP-1 receptor agonists can be utilized [99]. GLP-1 receptor agonists are providing more evidence of their potential to achieve the following: (1) correct vascular dysfunction, this being the first step of the atherogenic cascade; (2) target diabetic dyslipidemia by modulating different steps of cholesterol homeostasis; (3) dampen inflammation by reducing many pro-atherosclerotic cytokines; (4) inhibit platelet aggregation and thrombosis; (5) reduce endoplasmic reticulum stress; (6) regulate adiponectin and other adipokines; (7) reduce epicardial adipose tissue, and finally (8) transform plaque toward stability [99].

There is a large body of data showing that GLP-1 RAs exert pleiotropic cardiovascular actions, in addition to their canonical effects on glycemia, appetite and weight regulation.

### 4.5. Evidence on Subclinical Carotid Atherosclerosis, and the Role of Subclinical Atherosclerosis

We have demonstrated that the major predictor of MACCE was the presence of carotid intima–media thickening above 1.0 mm and the identification of carotid plaque (≥1.5 mm) on the initial assessment. When an individual patient presents with carotid atherosclerotic plaque at baseline, this is an important finding consistent with the individual predisposition to develop more atherosclerotic lesions in the future, despite initially normal angiograms. In the Cardiovascular Risk in Young Finns Study, the highest ASCVD incidence (11.0%) was found among those who were estimated to have a high risk (score2 ≥ 2.5% 10-year risk) and who showed a positive ultrasound scan for carotid plaque and/or high IMT (upper decile) in multivariate models [100].

In our former study, in patients diagnosed with significant ASCVD, evidence of carotid subclinical atherosclerosis was associated with a MACCE incidence of 18.6% in just a two-year follow-up period [101]. CIMT thickening above >1.3 mm (*p* < 0.001), diabetes (*p* = 0.018), and LDL-cholesterol > 3.35 mmol/L (*p* = 0.012) were associated with 1.27-fold, 1.14-fold and 1.15-fold event risk increases, respectively [101].

In the present study, despite the initial exclusion of significant (≥50%) arterial stenosis across four major vascular territories, approximately one in six patients experienced MACCE during the follow-up period. This highlights the limitations of a purely “obstructive-centric” diagnostic approach, and underscores the importance of subclinical markers and dynamic metabolic risk factors [102,103,104]. In addition, subclinical carotid atherosclerosis appears to be a very powerful indicator of multi-level atherosclerosis [105,106].

This suggests that even when arterial lumen reduction is minimal, the presence of plaque corresponds to an active, systemic atherosclerotic process. CIMT progression and non-obstructive plaques reflect the cumulative biological impact of risk factors on the arterial wall, which may not be captured by traditional risk scores alone [107,108]. This finding is consistent with a recent study suggesting that carotid plaque assessment provides significant incremental value in risk stratification, particularly in individuals initially classified as low- or intermediate-risk [109]. In a population of 5716 asymptomatic U.S. adults (mean age 68.9 years) who underwent examination by vascular ultrasound to quantify carotid plaque burden and by computed tomography for coronary artery calcium, the carotid plaque burden and calcium score were both significantly associated with all-cause mortality (fully adjusted trend HR—1.23; 95% CI—1.16–1.32; and HR—1.15; 95% CI—1.08–1.23), respectively (both *p* < 0.001), over a median 12.4-year follow-up [109].

Last but not least, ideally, a repeat carotid ultrasonography should be offered to the individual patients in whom the initial examination showed CIMT thickening and/or carotid plaque [107,108]. Carotid plaque progression was associated with an HR of 1.03 (95% CI—1.01–1.04 per absolute 10 mm^3^ change; *p* = 0.01) [109]. Attenuated CIMT progression might result in a reduced risk of MACCE by 75%, compared to individuals with evidenced CIMT/plaque progression [107]. Meanwhile, a reduction in carotid plaque progression by 0.01, 0.02, 0.03, and 0.04 mm/year was associated with a reduction in cardiovascular risk between 16% and 37% [108].

## 5. Conclusions, Future Perspectives and Closing Remarks

Our findings support the need for continued risk factor optimization, despite the initial exclusion of obstructive ASCVD. In this study, we have identified a new onset T2DM, initial LDL-cholesterol, and the presence of subclinical atherosclerosis as risk factors for MACCE.

Despite ruling out significant ASCVD with imaging work-ups, the watchful waiting policy, together with active risk factor modification, should be applied. Our study demonstrated that approximately 15% of patients would develop MACCE attributed to atherosclerosis progression to the stage of occlusive arterial disease.

Thus, patients with risk factors who are found to have “normal” or non-obstructive arteries should not be reassured and dismissed. Instead, the presence of subclinical atherosclerosis and markers of metabolic disease should trigger aggressive preventive measures [110,111,112,113]. In addition our findings discourage the discontinuation of anti-atherosclerotic medical treatment and less attentive atherosclerotic risk factors control against the individual patient’s false sense of security regarding cardiovascular event risk. This attitude is supported by the other researchers’ findings [41,56,81,114,115].

Instead, patients should remain on medical treatment with the aim of achieving optimal cardiovascular risk factors control. The recently-published multicenter study showed a strong evidence base for this strategy. The absence of five classic risk factors at age 50 was associated with more than a decade greater life expectancy than the presence of all five risk factors, in both sexes. Persons who modified hypertension and smoking in midlife had the most additional life years free of cardiovascular disease and death from any cause, respectively [41].

## 6. Study Limitations

Our present study has some limitations. First, it was a single-center, observational and retrospective study. Thus, multicenter and multi-ethnic prospective studies would be important. Additionally, more up-to-date biochemical data could be applied including lipoprotein (a) levels and genetic testing, which were unavailable at the time of the patients’ index hospitalization.

Although we have reported baseline medications such as statins, antiplatelet agents, hypoglycemic, and blood lowering agents, applications and changes to dose and regiment may have occurred that were not recorded in the present study. Furthermore, we have not reported blood pressure values due to low self-reporting by the patients, or inconclusive self-reporting. Additionally, the patients’ adherence to statin or blood-lowering therapy was not strictly monitored. However, we acknowledge that the effectiveness of lipid- and blood-lowering therapies makes them cornerstones of atherosclerosis attenuation and lowering the risk of MACCE.

The relatively low number of MACCEs (*n* = 30) may increase the risk of model instability; however, to minimize the risk of overfitting and maintain statistical reliability, we have strictly limited the multivariable Cox regression models to variables with a *p*-value less than 0.01 in univariate Cox analysis, resulting in only three variables entering a multivariate Cox regression analysis in each of the two models. This design achieved an events-per-variable (EPV) ratio of exactly 10; therefore, it adheres to the traditionally accepted standard threshold for survival analysis.

## Figures and Tables

**Table 1:** Baseline group characteristics.

Baseline Parameter	All Participants *N* = 188	MACCE (+) Group *N* = 30	No MACCE Group *N* = 158	*p*-Value
Age [years], mean (SD)	62.6 (8.75)	63.1 (9.24)	62.5 (8.68)	0.719
Male gender, *n* (%)	121 (64.4)	17 (56.7)	104 (65.8)	0.337
Female gender, *n* (%)	67 (35.6)	13 (43.3)	54 (34.2)	-
BMI [kg/m^2^], mean (SD)	27.42 (3.83)	27.5 (4.06)	27.4 (3.81)	0.8977
Normal body weight (BMI 20–24.9 kg/m^2^)	42 (22.3)	6 (20)	36 (22.8)	
Overweight or obese (BMI ≥ 25 kg/m^2^)	141 (75)	23 (76.7)	118 (74.7)	
Underweight (BMI < 20 kg/m^2^)	5 (2.7)	1 (3.3)	4 (2.5)	
Arterial hypertension, *n* (%)	138 (73.4)	23 (76.7)	115 (72.8)	0.659
T2DM, *n* (%)	39 (20.7)	6 (20.0)	33 (20.9)	0.913
Smoking, *n* (%)	67 (35.6)	14 (46.7)	53 (33.5)	0.169
Hiperlipidemia, *n* (%)	143 (76.1)	28 (93.3)	115 (72.8)	0.016
Number of risk factors, mean (SD)	2.77 (1.16)	2.97 (1.22)	2.73 (1.15)	0.317
Serum biochemical biomarkers				
Glucose [mmol/L], mean (SD)	5.65 (1.42)	5.73 (1.56)	5.64 (1.39)	0.754
HbA1c [%], mean (SD)	5.61 (0.30)	5.84 (0.31)	5.55 (0.30)	0.215
Triglycerides [mmol/L], mean (SD)	1.49 (0.87)	1.52 (0.76)	1.48 (0.89)	0.821
Total cholesterol [mmol/L], mean (SD)	5.26 (1.17)	5.49 (1.24)	5.21 (1.16)	0.239
LDL-cholesterol [mmol/L], mean (SD)	3.15 (0.97)	3.47 (1.11)	3.08 (0.92)	0.043
HDL-cholesterol [mmol/L], mean (SD)	1.37 (0.43)	1.35 (0.36)	1.37 (0.44)	0.818
Creatinine [µmol/L], mean (SD)	87.1 (23)	93.6 (32)	85.7 (23)	0.089
eGFR [ml/min/1.73 m^2^], mean (SD)	83.2 (23.4)	74.8 (29)	84.9 (23)	0.037
hs-CRP [mg/L], mean (SD)	2.86 (3.19)	3.34 (3.21)	2.75 (3.19)	0.344
Carotid ultrasonography (baseline)				
CIMT [mm], mean (SD)	0.991 (0.232)	1.160 (0.273)	0.958 (0.209)	<0.001
CIMT > 1.0 mm, *n* (%)	80 (42.6)	23 (76.7)	57 (36.1)	<0.001
Carotid plaque > 1.5 mm, *n* (%)	69 (36.7)	22 (73.3)	47 (29.7)	<0.001
LV EF [%], mean (SD)	61.0 (12.6)	59.1 (14.2)	61.5 (12.3)	0.357

Abbreviations: BMI, body mass index; CIMT, carotid intima–media thickness; HbA1c, glycated hemoglobin; HDL, high-density lipoprotein cholesterol; hs-CRP, high-sensitivity C-reactive protein; LDL, low-density lipoprotein cholesterol; LV-EF, Left Ventricular Ejection Fraction; SD, standard deviation; T2DM, type 2 diabetes mellitus.

**Table 2:** Predictors of MACCE in patients with initially ruled-out significant arterial obstructive disease in the univariate and multivariate Cox proportional hazard analysis.

Baseline Parameter	Univariate Cox HR (95% CI); *p* Value	Multivariate Cox HR (95% CI); *p* Value MODEL 1 *	Multivariate Cox HR (95% CI); *p* Value MODEL 2 *
Age [year]	1.01 (0.97–1.06); 0.63		
Male sex	0.67 (0.33–1.39); 0.28		
Hypertension	1.11 (0.49–2.49); 0.80		
Hiperlipidemia	4.89 (1.17–20.5); 0.03		
Baseline LDL-cholesterol (as a continuous variable)	1.04 (1.01–1.08); 0.025		
Baseline LDL-cholesterol > 3.2 mmol/L	3.21 (1.47–7.01); 0.003	2.19 (0.99–4.94); 0.05	2.90 (1.32–6.35); 0.008
Smoking	1.56 (0.76–3.20); 0.22		
T2DM (by onset, time adjusted):			
No T2DM (reference)	-		
Baseline T2DM	1.35 (0.39–3.76); 0.56		
New onset T2DM	36.89 (16.20–84.04); <0.001		
T2DM (all, time adjusted)	3.98 (1.94–8.16); <0.001		
Time-adjusted new onset T2DM	34.36 (15.78–74.82); <0.001	18.62 (7.97–43.50); <0.001	21.47 (9.33–49.40); <0.001
Initial carotid plaque presence	5.41 (2.10–10.97); <0.001	4.02 (1.72–9.36); 0.001	
Mean CIMT value > 1 mm	3.82 (1.70–8.58); 0.001		2.89 (1.27–6.59); 0.012
CIMT by quartiles			
Q1 (<0.825 mm) (reference)	-		
Q2 (between 0.825 and 0.982 mm) vs. Q1	1.76 (0.34–9.06); 0.50		
Q3 (between 0.983 and 1.332 mm) vs. Q1	3.69 (0.80–17.08); 0.10		
Q4 (≥1.333 mm) vs. Q1	6.30 (1.43–27.72); 0.015		

* model *p* value < 0.001. CI—confidence interval; CIMT—carotid intima–media thickness; HR—hazard ratio; MACCE—major adverse cardiac and cerebral events; Q1—first quartile; Q2—second quartile; Q3—third quartile; Q4—fourth quartile.

## Data Availability

Data are available upon reasonable request.
